# Evaluating the Efficacy and Safety of Patient “Virtual” Self-Removal of Ureteric Stents With Extraction Strings Following Ureterorenoscopy

**DOI:** 10.7759/cureus.97042

**Published:** 2025-11-17

**Authors:** Chaitya Desai, Piyush Sarmah, Raghuram Devarajan

**Affiliations:** 1 Urology, University Hospitals Coventry and Warwickshire NHS Trust, Coventry, GBR

**Keywords:** endo urology, renal stone surgery, self-removal, ureteric stent, ureteric stent removal, ureterorenoscopy (urs)

## Abstract

Introduction: Ureterorenoscopy (URS) is commonly followed by ureteric stent placement to maintain drainage and prevent complications. Stents with extraction strings allow for patient self-removal, potentially reducing hospital visits and healthcare costs. We evaluated the safety and efficacy of a *virtual* self-removal pathway, whereby patients removed their own stent at home and confirmed removal by sending a photograph to their consultant, or respective secretary, via email.

Methodology: A prospective review was undertaken of all patients who received a ureteric stent with an extraction string after URS at a UK tertiary center between 2016 and 2021. Patients were provided verbal and written instructions, including red-flag symptoms and details of the *virtual* confirmation process. Data collected included demographics, stone location, stent dwell time, method of removal, complications, and patient feedback.

Results: A total of 112 patients (median age 48.2 years, range 18-86; 46 (41%) were female and 66 (59%) male) met the inclusion criteria. Median stent dwell time was 4 (range 0-10) days. Ninety-nine patients (88.4%) successfully self-removed their stents at home with email confirmation, while 10 patients (8.9%) underwent removal by a healthcare professional. Complications occurred in 9 patients (8.0%), none exceeding Clavien-Dindo grade II. No adverse patient feedback was reported. Compared with published literature, complication rates and stent dislodgement were lower. The practice was associated with significant estimated cost savings by avoiding outpatient cystoscopy.

Conclusions: Patient self-removal of ureteric stents using extraction strings, with virtual confirmation, is a safe and effective strategy. It minimizes hospital visits, reduces healthcare costs, and empowers patients. Wider adoption of this model could standardize care pathways and support NHS sustainability targets. Further multicenter studies and randomized trials are warranted to validate these findings.

## Introduction

Ureterorenoscopy (URS) is a common operation utilized for visualizing and managing upper urinary tract pathology such as renal or ureteric calculi [1]. Following URS, stents with a ‘double J’ design are commonly placed within the ureter to maintain patency (reduce the sequelae of oedema or stricture formation from instrumentation) and encourage passage of small calculi fragments [2,3]. However, studies have cited potential side effects of ureteric stents such as urinary frequency, urgency, dysuria, hematuria, and flank pain [4-6]. These stent symptoms can lead to increased emergency department visits, additional analgesic medications, and the need for early stent removal - traditionally in an outpatient flexible cystoscopy clinic.

Many modern stents are manufactured with extraction strings attached to the stent’s distal end. These strings are made of fine suture material and, when placed, run out through the urethral meatus and can then be secured to the patient’s mons pubis (in women) or to the penis (in men). A 2018 systematic review by Oliver et al. showed that there is no overall increased morbidity or decrease in patient quality of life by utilizing stents with extraction strings [7]. Importantly, the use of extraction strings can avoid an invasive flexible cystoscopy procedure, and the patient can even remove it themselves at home. Thus, reducing the constraints on outpatient clinic bookings and preventing extended delays in stent removal. Studies have also shown that patients have a preference towards extraction strings as they feel less pain compared to flexible cystoscopy removal, and those patients in rural centers find the removal process more convenient than travelling long distances to reach a hospital [8-10].

During the COVID-19 pandemic, there was an increase in the implementation of virtual outpatient clinics, particularly across Urology as a specialty in the United Kingdom [11-14]. These clinics were typified by healthcare professionals (HCPs) using real-time communication technology such as telephone, video-calling, or live chat to run outpatient clinics with patients. These virtual clinics had the primary advantage of reducing hospital footfall during the pandemic for non-urgent cases, but also showed several additional benefits. A 2020 systematic review by Edison et al. reported direct cost savings for National Health Service (NHS) Trusts and reduced travel-related expenses for patients, with patients describing the experience as “safe, thorough, and professional” [15]. This travel reduction has an indirect beneficial impact on the environment, as currently the NHS is responsible for 5% of all UK road transport emissions [16].

The aims of the present study were twofold. The primary outcome was to evaluate the efficacy and safety of patients self-removing their stent at home and reporting the outcome by emailing a photograph of the removed stent as a practical and safe method of implementing this strategy.

## Materials and methods

*Virtual* patient self-removal: Our practice

A prospective review was conducted for patients who had stents with extraction strings placed following URS in a single NHS tertiary care urological center in the United Kingdom. The decision to leave a stent with a string was made by the consultant surgeon performing the procedure. Stents used were indwelling double *J* type (Boston Scientific, Marlborough, MA), with extraction strings attached to their distal ends. Following the URS, the stent would be in situ for a maximum of 10 days. Patients were given clear postoperative verbal instructions and a printed information sheet explaining how to self-remove the stent and specifying the exact postoperative day on which to do so. The information sheet crucially contained the consultant’s email address, where the patient was instructed to send a photograph of the removed stent as an attachment. Furthermore, patients were informed about red-flag signs to watch for, such as the string detaching from the stent in situ, and were advised on how to urgently contact the urology department.

Data collection

Prospective data collection occurred over a five-year period from 2016 to 2021. Inclusion criteria were patients undergoing URS for calculus removal who received a postoperative stent with an extraction string and had a maximum in-situ time of 10 days. Exclusion criteria were patients who did not receive a stent after URS. Additional exclusion reasons included the need for prolonged stent duration, ureteric trauma, and patient refusal. Data collected included patient demographics (gender and age), calculus location, preoperative stent placement, anatomical considerations, intraoperative complications, stent laterality, intended method and timing of stent removal (from the operation note), actual method of stent removal, number of postoperative days until stent removal, complications related to stent removal, and patient comments.

## Results

**Figure 1:**
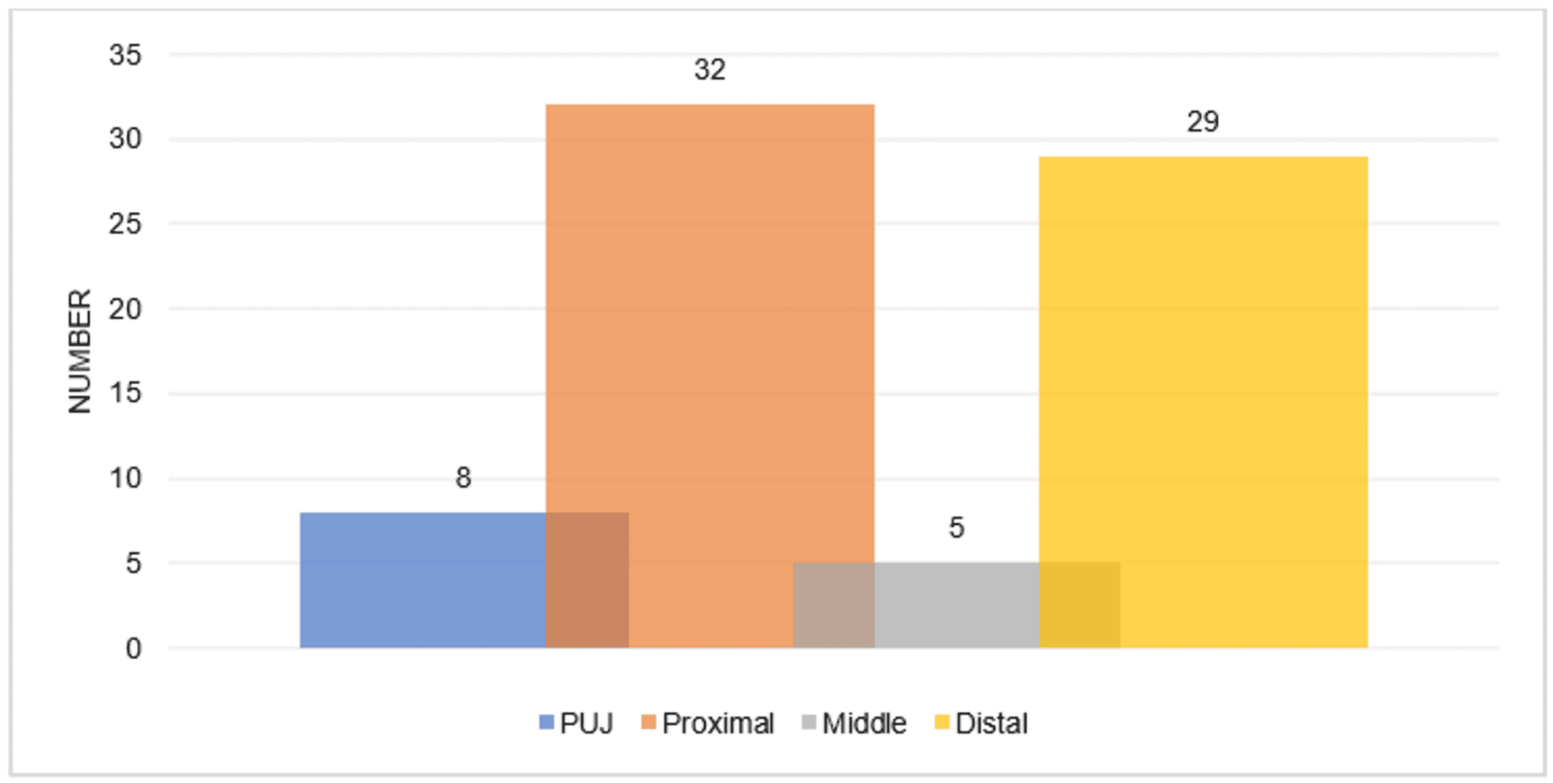
The anatomical position of ureteric calculi during ureterorenoscopy. PUJ, pelvi-ureteric junction

**Figure 2:**
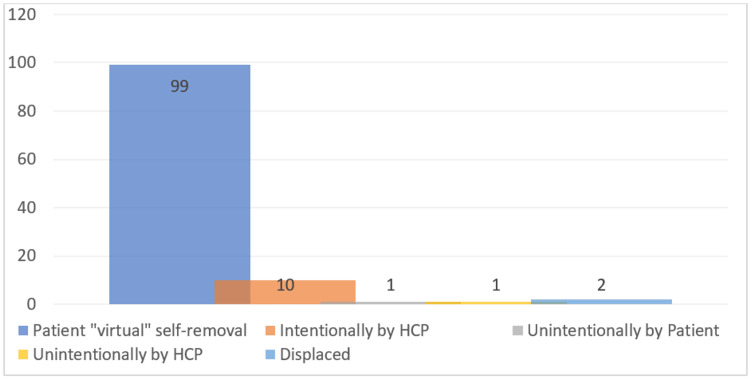
The method by which the ureteric stent was removed. HCP, healthcare professional

**Figure 3:**
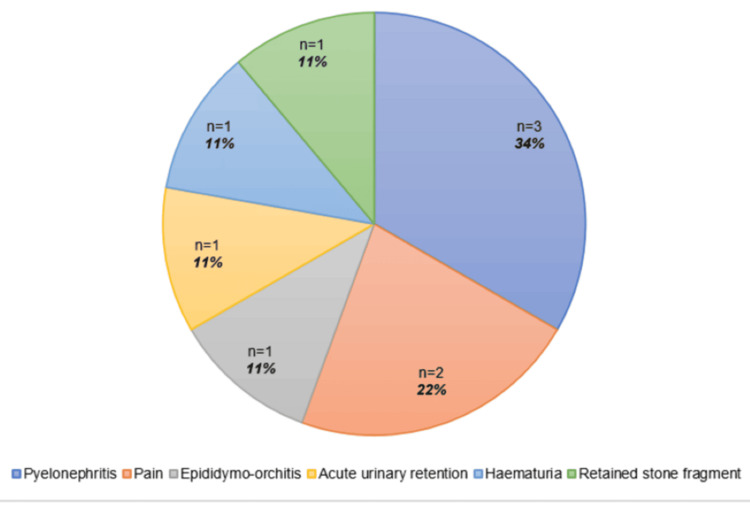
The complications identified postoperatively in study participants.

Over the five-year study period, a total of 112 patients were identified and met the inclusion criteria (Methods), based on a retrospective database maintained by the surgeons. From the sample, 46 (41%) were female and 66 (59%) were male, with a median age of 48.2 (age range: 18-86) years. Thirteen patients (12%) were pre-stented, and the rest had undergone primary URS procedures with subsequent stenting. Thirty-one patients (28%) underwent semi-rigid ureteroscopy only, and 81 patients (72%) underwent further flexible ureteroscopy procedures. Seventy-four patients (66%) had ureteric calculi, and Figure 1 highlights their anatomical location. Six patients (5%) had complex anatomy (e.g., horseshoe kidney, renal duplication). Stents were left in situ for a median duration of 4 (range: 0-14) days. The method of stent removal varied, but the vast majority of patients (99, 88%) performed self-removal of their ureteric stents at home, with virtual validation via email. The full breakdown of stent removal methods is shown in Figure 2. Nine patients (8%) experienced complications while the stents were in situ or post-removal, but none exceeded Grade 2 in the Clavien-Dindo classification system. Figure 3 illustrates the specific complications. No negative patient comments were recorded.

## Discussion

The literature regarding the efficacy and safety of ureteric stents with extraction strings was unexpectedly limited, and no studies reviewing a method of *virtual* self-removal in a UK population were found. This was unusual since stents with extraction strings are commonly used in the United Kingdom for short-term indications post-ureteroscopy and laser lithotripsy. The most extensive work and a reference point for this discussion was the systematic review by Oliver et al., which identified 483 patients with the removal of a stent via an extraction string [7]. Our prospective five-year study of consecutive UK patients adds to these findings by focusing on the specific methodology of how patients can confirm their stent self-removal to their urologists on a routine basis. Generally, our results show that stent self-removal using extraction strings in the appropriately selected patient cohort can reduce stent dwell times (from awaiting outpatient clinic appointments) with satisfactory complication rates and a favorable patient experience.

Safety is paramount when considering patient-driven interventions. Our study demonstrated a low rate of complications compared to the literature. The observed stent displacement rate of 0.89% (*n* = 1) was significantly lower than the 9.9% pooled rate reported by Oliver et al. [7]. Furthermore, none of the patients had a tear or break in their extraction strings. In terms of string securement, our practice involved a Tegaderm transparent film dressing to secure the distal string loop onto the mons pubis in females, or the penile shaft in males. The key to this technique was keeping laxity in the string loop to prevent unintentional breakage during underwear removal, sexual intercourse, or male erections. Another key factor that may have contributed to the lower rate of string complications is the information provided to patients during the perioperative period. Our practice included a locally designed patient information leaflet with photographs to show what a stent with an extraction string looks like, the advantages and potential risks of the extraction string, clear instructions for self-removal, and an email address of the surgeon for sending the eventual photograph of the removed stent. Anecdotally, patients expressed that this clear communication channel to their surgeon was an extremely useful tool for reassurance and troubleshooting during the ‘virtual’ post-operative period.

Our aim in this study was to have all participants undergo self-removal of their stents at home. However, we found that this was only the case in 99 patients (87%). The remainder of study participants included 10 patients (9%) whose stents were intentionally removed by a healthcare professional (HCP), 1 patient (1%) whose stent was unintentionally removed by an HCP, 1 patient (1%) who inadvertently removed the stent themselves, and 2 patients (2%) who experienced stent displacement. We do not consider intentional stent removals by an HCP (usually a urology nurse specialist) to be a failure in our practice. These removals were still performed by simply pulling on the extraction string and therefore avoided the need for a flexible cystoscopy procedure. However, there was an added cost of HCP time and potential for reduced patient convenience. For the one patient who had accidental removal of the stent by an HCP in a hospital setting, the reason is unclear in the medical notes. The literature describes successful patient self-removal rates ranging from 88% to 97% in a clinical trial setting [17,18]. Therefore, our results are within the accepted range in the literature.

In addition to low perioperative complication rates, self-removal of stents lessens the physical and financial strain on patients but also alleviates the financial burdens on a government healthcare service such as the NHS in the United Kingdom. For the 88.4% of patients who successfully self-removed their stents at home, hospital visits were avoided, reducing the risk of acquiring infections, particularly during the COVID-19 pandemic, and lowering associated travel expenses. Furthermore, a study based in a Leicestershire hospital in the United Kingdom found that each flexible cystoscopy and stent removal procedure costs approximately £900 (including staffing, equipment, equipment cleaning, and upkeep) [19]. If the financial projections from that study are matched to ours, then over five years, the 99 study participants who completed self-removal saved the department close to £90,000. Furthermore, this figure could rise further as our department utilizes disposable cystoscopes to complete the procedure, with each costing £135.23 per procedure [20].

In our hospital, discussions about the possibility of patients removing stents themselves typically take place in the clinic or on the wards in the first instance and then are re-checked on the morning of surgery during the consent process. Patients who strongly object to self-removal or have a significant clinical need for a postoperative indwelling stent do not receive a stent with an extraction string. Otherwise, the standard practice involves planning for self-removal of the stent. During our study period, overall patient-reported experiences were positive, as no negative feedback was received. Thorough preoperative discussions and the provision of written instructions in the form of a specific information leaflet upon discharge play crucial roles in ensuring a positive patient experience. Furthermore, based on our findings, extremes of age (ranging from 18 to 86 years) or gender did not emerge as factors influencing complication rates or patient experiences related to stent self-removal.

The *virtual* nature of confirming patient self-removal of ureteric stents proposed by our practice is novel in the literature. The low complication rates and no patient-reported concerns show that this method of managing patients with short-term ureteric stents post-URS has merit. The Getting It Right First Time (GIRFT) program has specifically highlighted the need to standardize the national care of urinary tract stones [21], and our promising local data may provide a foundation for national implementation of this *virtual* strategy. The rise of technology in supporting healthcare communication is evident, and the COVID-19 pandemic acted as a catalyst for its implementation across the NHS in the United Kingdom. Empowering patients to self-extract their own ureteric stent is effective and safe for most patients. Clear written and verbal communication before the day of surgery, and email as a method of follow-up, are paramount to facilitate this.

It should be acknowledged that the duration of stent indwelling in patients undergoing self-removal using a stent with an extraction string is likely shorter than in those scheduled for elective cystoscopic removal. This may be attributed to increased patient motivation to remove the stent promptly once clinically appropriate, as well as the avoidance of delays associated with hospital scheduling and cystoscopy waiting lists. However, this observation remains speculative, as our study did not formally assess or compare stent dwell times, nor did it demonstrate an explicit intention of temporality in conventional practice, where stents are typically removed via cystoscopy.

The limitations of this study include the design being limited to one hospital site, and we endeavor to build on a future multi-site project to further validate the evidence base for the use of stents with extraction strings. We appreciate that there was no control group for comparison used (i.e., stents without extraction strings) to evaluate data points such as indwelling times. The clinical outcomes and patient feedback were based only on up to 14 days after the postoperative period. However, longer-term outcomes of stents on extraction strings have not been investigated in this study and remain under-reported in the literature.

## Conclusions

The use of stents with extraction strings for patient self-removal is a safe and effective retrieval modality. With appropriate patient selection, this method can be *virtually* verified by the patient sending an e-mail photograph of the removed stent to the urological surgeon. A printed information leaflet detailing clear instructions is vital. This small prospective study has the potential to guide a future randomized controlled trial to further the understanding of this novel method for verifying patient self-removal of ureteric stents with extraction strings.
